# Cap-Assisted Direct Endoscopic Necrosectomy for Walled-Off Pancreatic Necrosis: A Case Report

**DOI:** 10.7759/cureus.86384

**Published:** 2025-06-19

**Authors:** Mustafa Süveran, Can Boynukara, Betul Piyade, Recep Cecen, Gürhan Sisman

**Affiliations:** 1 Gastroenterology and Hepatology, Acibadem Altunizade Hospital, Istanbul, TUR; 2 Internal Medicine, Acibadem University, Istanbul, TUR; 3 Internal Medicine, Marmara University, Istanbul, TUR; 4 Internal Medicine, Istanbul University-Cerrahpasa, Istanbul, TUR

**Keywords:** acute necrotizing pancreatitis, direct endoscopic necrosectomy, pancreatic disease, pancreatic necrosectomy, surgical endoscopy

## Abstract

Acute necrotizing pancreatitis is a severe and potentially life-threatening gastrointestinal disease. Walled-off pancreatic necrosis is a serious complication of it. Direct endoscopic necrosectomy (DEN) is considered the first-line therapy. Larger debridement through endoscopy may be both time- and cost-effective, in addition to facilitating faster recovery. Using an endoscopic cap to achieve more necrotic material could be an effective addition to the procedure. A 73-year-old female patient presented with severe abdominal pain, lethargy, and weight loss for the past three months. She was diagnosed with acute necrotizing pancreatitis complicated by an infected walled-off pancreatic necrosis. A total of three, one without and two with cap assistance, DEN procedures were performed. The mean duration of necrosectomy sessions was 52.3 minutes. Large amounts of necrotic material were removed using the endoscopic cap. The patient fully recovered and was discharged without any complications. She is healthy on the six-month follow-up visit. Cap-assisted DEN could be a time- and cost-effective treatment method due to its ability to enable larger debridement.

## Introduction

Acute pancreatitis is a gastrointestinal disease ranging from mild oedema of the pancreas to severe necrotizing pancreatitis. The annual incidence of acute pancreatitis is between 13 and 45/100,000 people [1]. Approximately 20% of cases develop necrosis; secondary infection occurs in 30% [2]. The mortality rate of acute necrotizing pancreatitis is 15% [3]. Transmural endoscopic drainage is considered an appropriate first-line treatment by the American Gastroenterological Association [4]. A randomized control trial detected that endoscopic and surgical approaches for the treatment of infected necrotizing pancreatitis were equally successful in reducing major complications or death. Additionally, the rate of pancreatic fistulas and duration of hospital stay were lower in the endoscopic intervention group [5]. In high-risk and very high-risk patients, direct endoscopic necrosectomy (DEN) is associated with reduced death rates compared to open necrosectomy [6].

During a DEN session, the maximum possible necrotic material should be removed. Large debridement could facilitate the healing process and the formation of granulation tissue. Furthermore, they could lead to fewer and shorter procedures, as DEN requires multiple sessions to completely debride the necrotic cavity. However, there has not been any development regarding the increase of tissue acquisition done by endoscopic instruments [5]. The utilization of different endoscopic instruments such as endoscopic caps, graspers, snares, and a combination of them could allow for a large debridement. The closed space of the endoscopic cap enables the suction of the necrotic material as well as allowing other endoscopic instruments through its lumen. Herein, we report a case of a patient with acute pancreatitis complicated by walled-off pancreatic necrosis (WOPN) and infection, who underwent three cap-assisted DEN procedures.

## Case presentation

**Figure 1:**
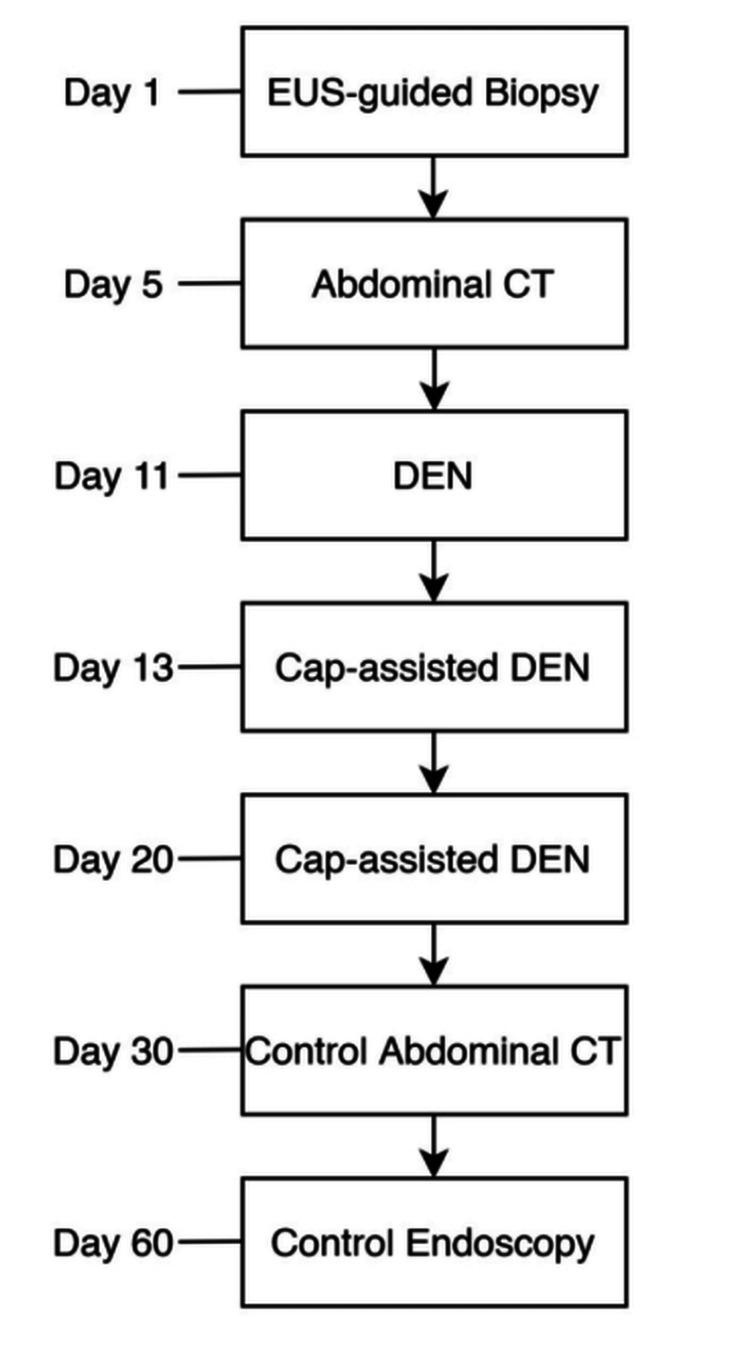
Planned patient care timeline. EUS: endoscopic ultrasound; CT: computed tomography; DEN: direct endoscopic necrosectomy.

In January 2021, a 73-year-old Caucasian female patient presented with severe abdominal pain, lethargy, and a 20 kg weight loss over the past three months. The patient was from Russia, and she had been diagnosed with acute pancreatitis with an infected WOPN there. However, due to technical reasons, she was referred to our clinic. The patient reported social drinking and no smoking. Generalized abdominal tenderness was present in the physical examination. The planned patient care is shown in Figure 1. The initial laboratory results showed decreased haemoglobin (11.1 g/dL, normal range: 11.9-14.9 g/dL), mean corpuscular volume (79.3 fL, normal range: 82.3-94.6 fL), and elevated C-reactive protein (CRP; 13.54 mg/dL, normal range: <0.5 mg/dL) (Table 1). Given the patient’s history, physical examination, laboratory results, previous endoscopy, and imaging findings, acute necrotizing pancreatitis complicated by an infected WOPN and microcytic anaemia was confirmed.

**Table 1:** Reference intervals of the laboratory tests. CRP: C-reactive protein.

Laboratory test	Laboratory values (before intervention)	Laboratory values (after antibiotherapy and intervention)	Reference interval
Leukocyte number	15.8x10^3^/uL	6.3x10^3^/uL	4.06-10.6x10^3^/uL
Haemoglobin	11.1 g/dL	12.8 g/dL	13.0-18.0 g/dL
Mean corpuscular volume	79.3 fL	80 fL	80.0-100.0 fL
Thrombocyte number	186x10^3^/uL	197x10^3^/uL	150-439 10^3^/uL
Serum creatinine	1.23 mg/dL	1 mg/L	0.7-1.3 mg/dL
Blood urea nitrogen	37 mg/dL	17 mg/L	6-20 mg/dL
CRP	13.54 mg/L	5.17 mg/L	<0.5 mg/L

**Figure 2:**
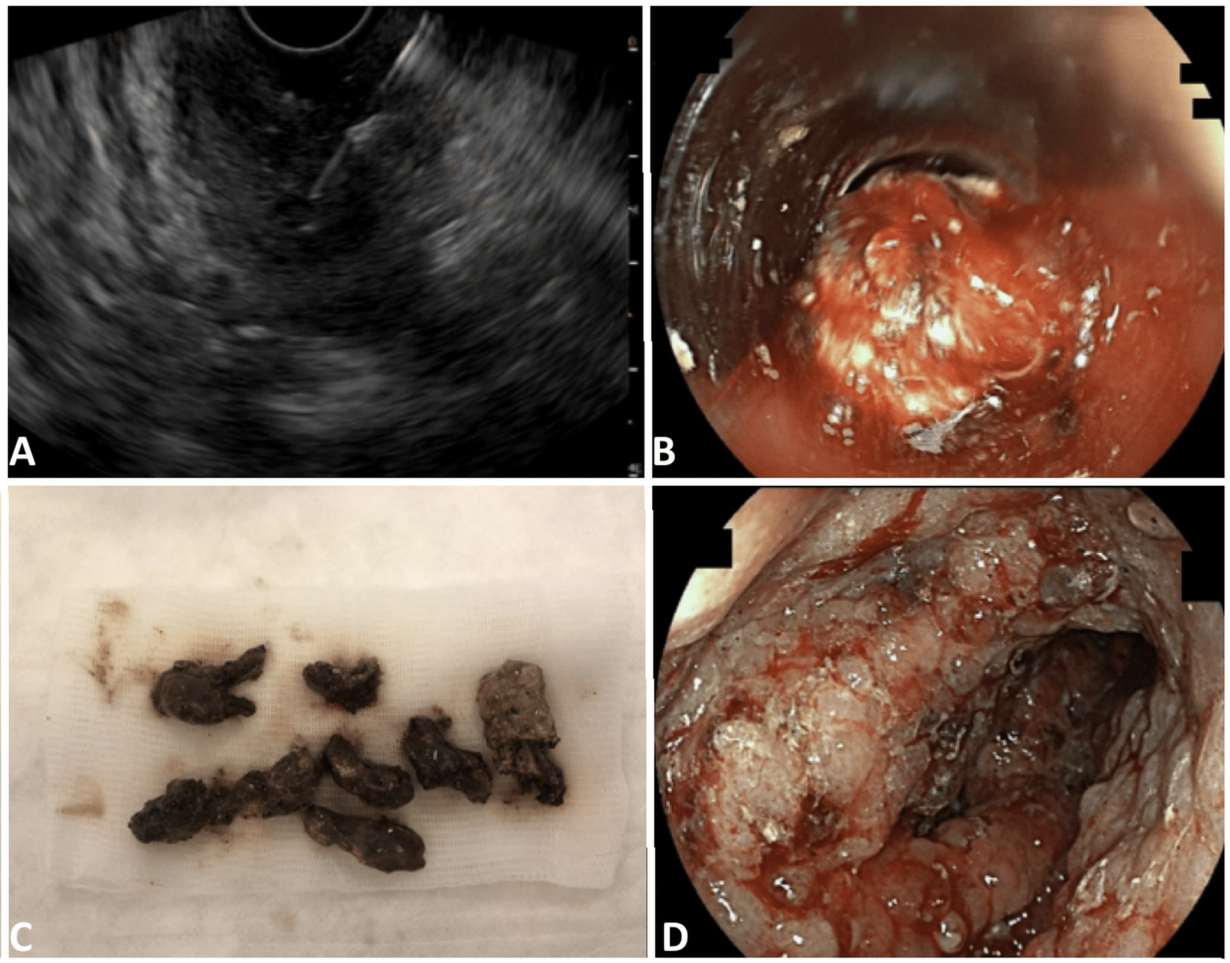
Images of the endoscopic procedures. (A) EUS-guided image of the WOPN at the initial evaluation measured as 46x47 mm. (B) Visualization through the cap. (C) Necrotic tissue removed with cap-assisted endoscopy. (D) The necrotic cavity at the end of the final necrosectomy. EUS: endoscopic ultrasound; WOPN: walled-off pancreatic necrosis.

Upper gastrointestinal endoscopy with endoscopic ultrasonography-guided biopsy from the WOPN was planned to evaluate the upper gastrointestinal system and determine the infectious agent (Video 1). The greater curvature side of the stomach was oedematous and inflamed. The WOPN was detected in the posterior wall of the stomach (Figure 2). Additionally, the biopsy results revealed *Escherichia coli* in the necrosis. The patient was hospitalized and started on intravenous imipenem/cilastatin 500 mg four times a day for four days. The patient’s CRP was in a decreasing trend, and her last CRP on the final day of the intravenous antibiotic treatment was 5.17 mg/dL (Table 1). The patient was discharged from the hospital with a prescription for oral amoxicillin/clavulanic acid 1000 mg three times a day for seven days.

**Video 1:** EUS-guided biopsy, standard DEN, and cap-assisted DEN procedures. EUS: endoscopic ultrasound; DEN: direct endoscopic necrosectomy.

**Figure 3:**
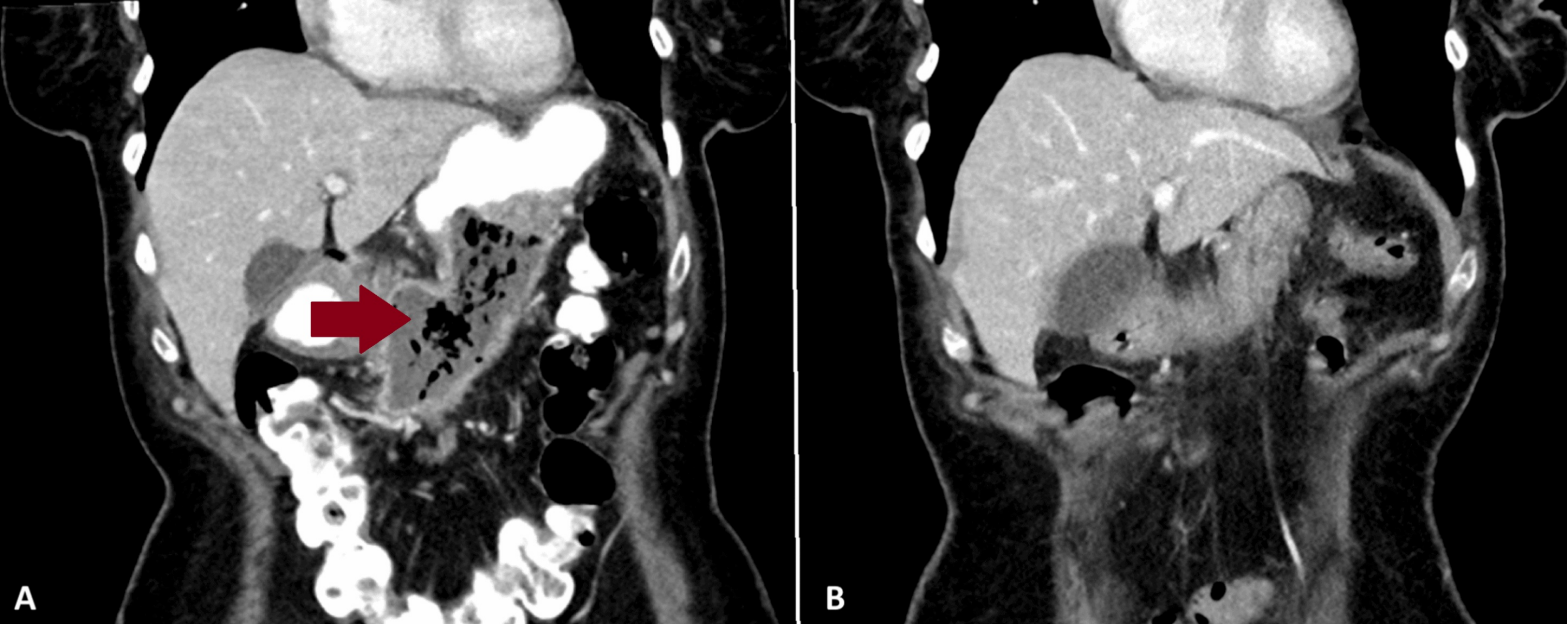
Abdominal CT images. (A) The initial abdominal CT with oral and IV contrast demonstrating peripancreatic necrosis and gas infiltration (arrow). (B) The control abdominal CT with IV contrast displaying no peripancreatic necrotic collections. CT: computed tomography; IV: intravenous.

Four days later, an abdominal computed tomography (CT) scan with oral and intravenous contrast detected an abscess measuring 44x83 mm with thick and enhancing walls containing gas, extending from the anterior corpus of the pancreas to the hilum of the spleen. Additionally, a second collection area measuring 25x17 mm was detected, expanding caudally from the uncinate process of the pancreas (Figure 3).

A standard DEN was performed six days after the abdominal CT (Video 1). The necrotic area in the posterior wall of the stomach was observed to be fistulated to the stomach. A guidewire was inserted into the necrosis, and a 2-cm self-expandable metallic stent (SEMS) (Micro-Tech, Nanjing, China; REF No.: NST33-544-12.020) was advanced over the guidewire and placed into the necrotic area through the artificial lumen. The necrotic area was visualized (Figure 2), and necrosectomy was performed using an endoscopic snare (20 mm Rotatable Snare, Boston Scientific, Massachusetts, USA; Catalog No.: M00561830), retrieval snare net (ROTH NET® Standard Foreign Body Retriever, STERIS, Ohio, USA; Product Code: 00711050), and tripod grasper (Polygrab™ Tripod, Olympus, Tokyo, Japan; Model No.: FG-600U). The session lasted 42 minutes.

A cap-assisted DEN was planned for two days after (Video 1). A sterilized transparent endoscopic cap (Model Number: DH28-GR; 15.2 mm diameter with the endoscope applied; Fujifilm, Minato, Tokyo, Japan) was placed onto the camera side of the endoscope (Figure 2). The necrosectomy was started with an endoscopic retrieval snare net and tripod grasper, and the necrotic area was profusely irrigated with 0.9% NaCl. Afterwards, the cap was placed, and the procedure continued (Figure 2). It allowed for the suction of a great amount of necrotic debridement (Figure 2). The SEMS was displaced by the cap during the procedure, and it was removed. Necrosectomy with endoscopic instruments and cap is continued. A major part of the necrotic cavity was debrided. Lastly, a nasojejunal tube was placed to feed the patient to prevent contamination in the stomach. The session lasted 87 minutes.

A second cap-assisted DEN was planned one week later (Video 1). The nasojejunal tube was removed. The necrotic tissue was completely debrided using an endoscopic retrieval snare net and tripod grasper and irrigated with 0.9% NaCl (Figure 2). The session lasted 28 minutes.

Nine days later, a control abdominal CT scan with intravenous contrast detected significant regression of the peripancreatic necrotic collections (Figure 3). One month later, a control endoscopy was performed, and spontaneous total closure of the artificial lumen was detected. The patient’s CRP was 0.08 mg/dL. The next follow-up visit was planned for six months later.

## Discussion

Cap-assisted DEN is a newly emerging safe choice of treatment in terms of having the same incidence of bleeding and perforation as video-assisted retroperitoneal debridement and reduced death rates compared to open necrosectomy in high-risk patients [2,7,8]. Additionally, by avoiding general anaesthesia and laparotomy or lumbotomy, less inflammatory burden is reflected onto the body, and the number of early and late complications such as adhesions, fistulas, hernias, and new onset of organ failure could be reduced. Moreover, without general anaesthesia, the potential negative effects on critically ill patients are prevented [8]. Furthermore, endoscopy provides better cosmetic results than other treatment methods by avoiding abdominal incisions. Lastly, combining endoscopic instruments allows for a large debridement and potentially shorter and fewer necrosectomy sessions.

One of the limitations of DEN is that some acute complications, such as perforations and major bleedings, could be difficult to manage endoscopically. More importantly, DEN is an advanced intervention. It requires a team of experienced interventional endoscopists, interventional radiologists, and hepatopancreatobiliary surgeons for treatment and complication management. Due to these requirements, endoscopic transgastric pancreatic necrosectomy procedures could only be performed in centres with multidisciplinary expertise [8].

DEN requires multiple sessions to completely debride the necrotic cavity. A larger debridement of the necrosis might shorten the number and duration of endoscopy sessions. A systematic review by van Brunschot et al. reported an average of four sessions for complete necrosectomy [8]. In this paper, three sessions were performed. A multicenter study reported that the mean duration of DEN for WOPN was 69 minutes [9].

In this case report, the mean duration for the three necrosectomy sessions was 52.3 minutes. The no-cap-assisted session was 42 minutes long, and the average was 57.5 minutes. Albeit one patient, it could be claimed that cap assistance expedites the duration of the necrosectomy procedures by 17%.

A larger debridement achievement was attempted in this patient by placing a sterilized endoscopic cap on the endoscope. The cylindrical design of the endoscopic cap enabled the suction of the endoscope to focus on the necrosis. The cap allowed us to remove a great amount of necrosis (Figure 2C). During the first cap-assisted session, the cap displaced the SEMS. Similarly, in a case series by Puri et al., the cap displaced the lumen-apposing metal stents in half of eight patients [2]. Therefore, large necrotic material should be carefully removed during a cap-assisted DEN. A large necrotic material also lodges the cap, an endoscopic instrument such as a grasper for forceful expulsion.

Three DEN sessions, the last two cap-assisted, were performed to treat the WOPN. No complications were observed, and the patient is expected to be in good health at the six-month follow-up visit. Cap-assisted DEN could be a safe method for treating WOPN.

The major limitation of this paper is that it is a case report describing a single patient and a single endoscopist who performed the procedures. Therefore, it lacks generalizability due to the nature of case reports. Further prospective studies on the effects of using single/combined endoscopic instruments on DEN with control groups must be conducted to maximize the effectiveness of this treatment.

## Conclusions

Cap-assisted DEN demonstrates significant potential as a safe and effective therapeutic approach for WOPN. This case report highlights its advantages, including reduced procedural duration (with cap-assisted sessions averaging 57.5 minutes compared to 42 minutes without), enhanced debridement capacity through endoscopic cap utilization, and avoidance of complications such as perforation, bleeding, or stent displacement when performed meticulously. The design of the cap facilitates targeted necrosis removal, potentially lowering the total number of required sessions while maintaining patient safety. However, the technical complexity of DEN necessitates a coordinated, multidisciplinary team and limits its adoption to specialized centres. The single-patient, single-operator design of this report remains constrained by the generalizability of the findings. Future prospective studies comparing cap-assisted versus non-assisted DEN, evaluating cost-effectiveness, and standardizing protocols for instrument use (e.g., cap-grasper combinations) are critical for optimizing outcomes. Despite these challenges, cap-assisted DEN represents a promising minimally invasive alternative to surgical necrosectomy, particularly in high-risk populations, warranting broader validation through controlled trials.
